# Different roles of elevational and local environmental factors on abundance‐based beta diversity of the soil Enchytraeidae on the Changbai Mountain

**DOI:** 10.1002/ece3.4913

**Published:** 2019-02-10

**Authors:** Xiaoming Jiang, Jing Chen, Zhicai Xie

**Affiliations:** ^1^ CAS Key Laboratory of Aquatic Biodiversity and Conservation, Institute of Hydrobiology Chinese Academy of Sciences Wuhan China; ^2^ State Key Laboratory of Eco‐hydraulic in Northwest Arid Region of China Xi'an University of Technology Xi'an China; ^3^ College of Life and Science Zao Zhuang University Zaozhuang China

**Keywords:** abundance‐based beta diversity, Changbai Mountain, elevational gradient, environmental sorting, soil potworm, substitution component

## Abstract

The elevational alpha biodiversity gradient in mountain regions is one of the well‐known ecological patterns, but its beta diversity pattern remains poorly known. Examining the beta diversity and its components could enhance the understanding of community assembly mechanism. We studied the beta diversity pattern of the soil enchytraeids along a distinct elevational gradient (705–2,280 m) on the Changbai Mountain, the best‐preserved mountain in northeastern China. The overall abundance‐based community dissimilarity was relatively high (ca. 0.70), largely due to the balanced‐variation component (85%). The overall dissimilarity and its balanced‐variation (substitution) component were related to both local environmental heterogeneity and elevational distance, with the environmental relationships being stronger. In contrast, the abundance‐gradient (subsets) component was not related to the two gradients. The same important spatial and environmental variables were detected in structuring overall dissimilarity and substitution component, different from that in subsets component. Variation partitioning analysis showed that environmental control played a more important role than spatial (vertical and horizontal) factors in structuring the patterns of overall beta diversity and its two components. The predictive power of multivariate analysis was higher for the substitution component (nearly 50%) and overall dissimilarity (35%), but much lower for subsets components (<4%). These findings implied that abundance‐based beta diversity patterns of the soil enchytraeids were the results of different ecological processes (e.g., environmental sorting and dispersal limitation), operating in the two antithetic components. Our study showed the substitution and loss of individuals reflecting different ecological processes and highlights the importance of partitioning beta diversity in assessing biodiversity patterns and their causes.

## 1 INTRODUCTION

Mountain areas support a high level of biodiversity, and some of them have long been the hotspot of biodiversity (Huber, Bugmann, & Reasoner, 2006; Qian, Ricklefs, & White, 2005). As the latitudinal biodiversity gradient, the elevational biodiversity gradient in these ecosystems is one of the well‐known ecological patterns for ecologists (Qian, Hao, & Zhang, 2014; Sanders, Lessard, Fitzpatrick, & Dunn, 2007). The elevational environment gradient is usually much steeper than the latitudinal gradient, for example, a temperature shift of a 100‐m vertical interval is approximately equal to that of a 100‐km latitudinal interval in the temperate zone (Crimmins, Dobrowski, Greenberg, Abatzoglou, & Mynsberge, 2011). Therefore, mountains with a distinct elevational gradient are deemed to be ideal arenas for examining biodiversity patterns due to the stronger response to the greater gradient of climatic or environmental conditions (Willis et al., 2010) and an avoidance or reduction of lags in community shifts for suitable habitats during climate change (Bertrand et al., 2011). Up to date, such elevational biodiversity patterns have been inspected for many taxa across different temporal and spatial scales (Rahbek, 2005; Shen et al., 2014; Wang et al., 2012).

Even though the elevational biodiversity gradients have been extensively investigated so far, their one key component, the beta diversity pattern, is still poorly known compared to the alpha diversity pattern (Teittinen, Kallajoki, Meier, Stigzelius, & Soininen, 2016; Wang et al., 2012). Beta diversity represents the heterogeneity in assemblage composition between localities and has been growing as a central issue in biodiversity studies (Legendre & Legendre, 1998; Whittaker, 1972). In recent decades, ecologists have paid more attention to beta diversity studies since it could provide useful information in understanding of different processes in species coexistence and community assembling (e.g., Anderson et al., 2011; Heino, Melo, & Bini, 2015). However, how to define and quantify beta diversity is still under debate, and the existing studies revealed that it is a multifaceted concept, and dozens of metrics based on incidence or abundance data have been developed to quantify the beta diversity in recent decades (Anderson et al., 2011; Barwell, Isaac, & Kunin, 2015).

Recently, new measurement approaches were proposed by ecologists, showing that overall beta diversity can be partitioned into two additive components: the turnover and nestedness‐resultant components, representing two different ecological processes driving the dynamics and behavior of a community (Baselga, 2010; Carvalho, Cardoso, & Gomes, 2012; Podani & Schmera, 2011). Species turnover reflects the species replacement from one locality to the next and may be driven by species sorting, biological reactions, and historical constraints (Qian et al., 2005). In contrast, when a poorer assemblage is a subset of a richer assemblage, the community overall dissimilarity consists completely of its nestedness component, which may link species loss to ecological processes such as species thinning and disaggregation (Gaston & Blackburn, 2000). Thus, the turnover and nestedness represent complementary processes in generating community variations of interest for assessing biodiversity patterns and exploring their causes (Baselga, 2010, 2013).

It has already been proven that the partitioning of the two fractions based on incidence data could promote the understanding of biotic patterns and their causes (Ding, Jiang, Xie, & Brosse, 2017; Leprieur et al., 2011; Villéger, Grenouillet, & Brosse, 2014). Recently, such partitioning has been expanded to abundance‐based beta diversity, namely balanced changes in abundance (the substitution of individuals by the same number of individuals of a different species) and abundance gradients (the assemblage with lower abundance is a subset of the assemblage with higher abundance), respectively (Baselga, 2013, 2017).

Both spatial variables (latitude, longitude, and elevation) and environmental filters can jointly affect the pattern of beta diversity (Anderson et al., 2011; Boieiro et al., 2013; Wang et al., 2012). Disentangling their roles on structuring biotic assemblages has recently become one of the immediate areas in biodiversity researches involving the concept of metacommunity. However, these studies mostly focused on the relative roles of spatial and environmental factors in driving overall dissimilarity, and few studies addressed this issue in regard to its turnover (or substitution) and nestedness (or subsets) components (Boieiro et al., 2013; Heino & Tolonen, 2017). Nevertheless, examining the drivers of the two additive components of beta diversity separately would be markedly helpful in understanding the metacommunity dynamics and revealing the mechanisms of assemblage variations (Baselga, 2013).

The soil enchytraeids (potworms) are a taxonomically and ecologically important group of soil mesofauna and often dominate the soil communities, especially in cold and wet, organic soil (up to 97% in terms of biomass, Briones, Ineson, & Heinemeyer, 2007). Potworms play key ecological roles in the soil compartment of ecosystems, such as modifying the soil structure, promoting the decomposition process and nutrient cycling (Cole, Bardgett, Ineson, & Adamson, 2002). Furthermore, they are particularly sensitive to temperature and moisture changes and thus considered as excellent indicators of global climate change and human disturbances (Briones et al., 2007; Holmstrup et al., 2012). A number of studies highlight pronounced effects of elevation or climate‐related variables on abundance and species composition of soil enchytraeids (e.g., Bauer, 2002; Schlaghamerský et al., 2014; Kõlli, Graefe, & Tamm, 2015), but knowledge of enchytraeid beta diversity patterns along elevational and local environmental gradients remains limited. Examining how elevational and local environmental factors drive the beta diversity patterns of enchytraeids, therefore, is not only important for the understanding of underlying mechanisms determining the composition of soil fauna, but also has great potential to increase our knowledge of how species assemblages change in response to projected global climate change.

The Changbai Mountain, being the highest mountain in northwest China and one of the well‐conserved ecosystems on Earth, represents a wide range of soils, vegetation, and climate conditions, with distinctly elevational distributions. This region supports a diverse and distinct soil fauna, from bacteria to eukaryotic macroorganisms (Shen et al., 2014). The local soil enchytraeid fauna is also rich in species, with many rare species and some endemic to this mountain (Chen, Xie, & He, 2006; Lian, Chen, Xiong, Jiang, & Xie, 2011; Shen, Chen, & Xie, 2012). The Changbai Mountain thus provides an ideal opportunity for examining natural enchytraeid diversity patterns along an elevational gradient. In this study, we first calculated the contribution of balanced changes in abundance (i.e., substitution) and abundance‐gradient (i.e., subsets) components to overall abundance‐based beta diversity of soil enchytraeids, along an elevational gradient from the hardwood forest habitat at the foot to the summit tundra of Changbai Mountain. Then, we explored the relationships between abundance‐based beta diversity (overall dissimilarity and its two components) and elevational or environmental distance (i.e., testing the distance decay of community composition along elevational or environmental gradients). We also evaluated the relative importance of spatial (vertical and horizontal) and environmental factors for patterns of overall beta diversity and its two components.

## 2 MATERIALS AND METHODS

### 2.1 Study area and sites

Data came from our previous work on 18 sites along the northern slope of the Changbai Mountain (Lian et al., 2011). Being the best‐preserved mountainous ecosystem in China, the Changbai Mountain is located on the border between China and North Korea and is a cone‐shaped mountain with its highest peak of 2,749 m. The 18 sites were distributed in the Changbai Mountain Natural Reserve (CMNR) and adjacent hardwood forest (0.8 km outside the nature reserve), with an elevational gradient from 705 m to 2,280 m. Along the vertical gradient on the north slope, we selected the sites subject to minimal anthropogenic disturbances in five forest types: hardwood forest (705–719 m, three sites); mixed forest (772–998 m, four sites); spruce–fir forest (1268–1693 m, four sites); birch forest (1753–1995 m, three sites); and tundra (2002–2280 m, four sites).

### 2.2 Enchytraeids and environmental data collection

On June 8, 2008, we sample three quantitative replicates using a split soil corer (area 20 cm^2^ and ca. 15 cm in depth, at ca. 7‐m intervals along a 20‐m transect) at each site. All samples were transported to the laboratory, and enchytraeids were extracted using a standard wet funnel extracting device (O'Connor, 1962). Specimens were examined while alive and identified to the lowest taxonomical level (majority to species, minority to genus level according to Lian et al., 2011) and counted.

A number of spatial and local environmental variables were simultaneously measured at each site. Altitude, latitude, and longitude were measured by GPS (Magellan 315). Twelve local environmental variables, including the soil and air temperatures (using a thermometer), and vegetation type, depth of litter, pH, soil moisture, organic matter content (550°C, 12 hr), content of K (by emission flame spectrometry), content of calcium (EDTA titration method), organic carbon (potassium dichromate–sulfuric acid oxidation method), organic nitrogen (Kjeldahl), and total phosphorus (Na_2_CO_3_–molybdenum–antimony anti‐spectrophotometric method) were also measured in the laboratory according to the soil and agro‐chemistry analytical methods of China (Lu, 1999).

### 2.3 Statistical analysis

To analyze abundance‐based assemblage dissimilarities, pairwise dissimilarities were calculated for the enchytraeid assemblages using the approach suggested by Baselga (2013). In this procedure, the Bray–Curtis dissimilarity index values (*d*_BC_) were decomposed into two additive components accounting for the balanced variation in abundances (*d*_BC‐bal_) and abundance gradients (*d*_BC‐gra_). The *d*_BC‐bal_ describes the variation in species density by keeping the overall species density constant (Baselga, 2013). This component is equivalent to species replacement in incidence‐based patterns, as the individuals are substituted by the same number of individuals of different species from site to site, that is, it corresponds to true species turnover. The *d*_BC‐gra_ describes the decrease or increase in species density from one site to the other with the overall species density declining or increasing, respectively. The *d*_BC‐gra_ is analogous to species nestedness in incidence‐based patterns, as some individuals are lost from one site to the other without any substitution in which an assemblage is a subset of another (Baselga, 2013, 2017).

Next, we regressed the pairwise overall dissimilarity (*d*_BC_), and its two‐component (*d*_BC‐bal_ and *d*_BC‐gra_) matrices against the elevational and environmental distances between sites, and calculated the significance of the Pearson correlations using Mantel tests on 9,999 permutations (Nekola & White, 1999). Prior to Mantel tests, the pairwise distance matrices of the elevational and environmental data were separately constructed using Euclidean distance. We used the Bio‐Env analysis to produce the best environmental distance matrix, and three variables (soil temperature, litter depth, and organic carbon) were selected in this process. Based on normalized environmental variables, this procedure tests all possible combinations of environmental variables and can provide information that shows the strongest correlation between biotic dissimilarity and environmental distance matrix. Prior to the Bio‐Env and subsequent analysis, two variables (air temperature and vegetation type) highly correlated to elevation (Pearson's *r* > 0.90) were excluded. In addition, we also ran partial Mantel tests based on three (biotic, elevational, and environmental) matrices to determine the pure effects of local environmental factors and elevation on *d*_BC_, *d*_BC‐bal_, and *d*_BC‐gra_.

Finally, we used distance‐based redundancy analysis (dbRDA) (Anderson, Gorley, & Clarke, 2008) to partition the contributions of the spatial (elevational and horizontal distance) and environmental variables to the overall dissimilarity (*d*_BC_), and its two components (*d*_BC‐bal_ and *d*_BC‐gra_). In this procedure, principal coordinates of neighbor matrices (PCNM; Borcard & Legendre, 2002) was first computed across the vertical (elevation) and the horizontal locations following Wang et al. (2012). The PCNM quantifies the spatial configuration of sample units based on principal coordinates of a truncated (nearest neighbors only) among‐sample distance matrix. The resulting PCNM axes with positive eigenvalues are used as spatial components in constrained ordination analysis (Gilbert & Bennett, 2010). We used the “pcnm” function in the vegan software package to generate the spatial PCNM axes for this analysis and retained 9 PCNM axes (PCNM1–PCNM9) with positive eigenvalues. Environmental factors and spatial filters were selected for further analyses, using forward selection (*p* < 0.10) based on 9,999 permutations. Prior to dbRDA, highly correlated independent variables (Pearson's *r* > 0.90) were removed, and the remaining ones were log(*x* + 1) transformed if they violated normality assumption. To examine the relative importance of environmental and spatial factors in explaining variation in beta diversity, we used a variation partitioning approach (Legendre & Legendre, 1998). In this analysis, the total percentage of variation explained by dbRDA is decomposed into pure and shared contributions of two sets of predictors (i.e., environmental and spatial factors). We report adjusted *R* ^2^ of pure and shared contributions of the spatial and environmental factors from the constrained ordinations, because of their impartiality and high recommendation in previous studies (Peres‐Neto, Pierre, Stéphane, & Daniel, 2006).

We ran all the analyses in R (R Development Core team, 2015), including calculating dissimilarity matrices in *betapart* package, and Mantel test, partial Mantel test, and dbRDA in *vegan* package.

## 3 RESULTS

The mean pairwise enchytraeid dissimilarities were 0.691 (*SD* = 0.170) for the Bray–Curtis (*d*_BC_) matrix, 0.605 (*SD* = 0.229) for the balanced‐variation (*d*_BC‐bal_) matrix, and 0.086 (*SD* = 0.100) for the abundance‐gradient (*d*_BC‐gra_) matrix. The mean relative contribution of the *d*_BC‐bal_ and *d*_BC‐gra_ to total dissimilarity was 85.0% and 15.0%, respectively (Table 1).

**Table 1:** Mean value, *SD*, and range (Min and Max) of Bray–Curtis dissimilarity (*d*_BC_), balanced variation (*d*_BC‐bal_), abundance gradient (*d*_BC‐gra_), and contribution of *d*_BC‐bal _(*p*_substitution_) and *d*_BC‐gra_ (*p*_subset_) to total dissimilarity

	Mean	*SD*	Min	Max
*d*_BC_	0.691	0.170	0.226	1
*d*_BC‐bal_	0.605	0.229	0.045	1
*d*_BC‐gra_	0.086	0.100	0	0.513
*p*_substitution_	0.850	0.195	0.117	1
*p*_subset_	0.150	0.195	0	0.883

**Figure 1:**
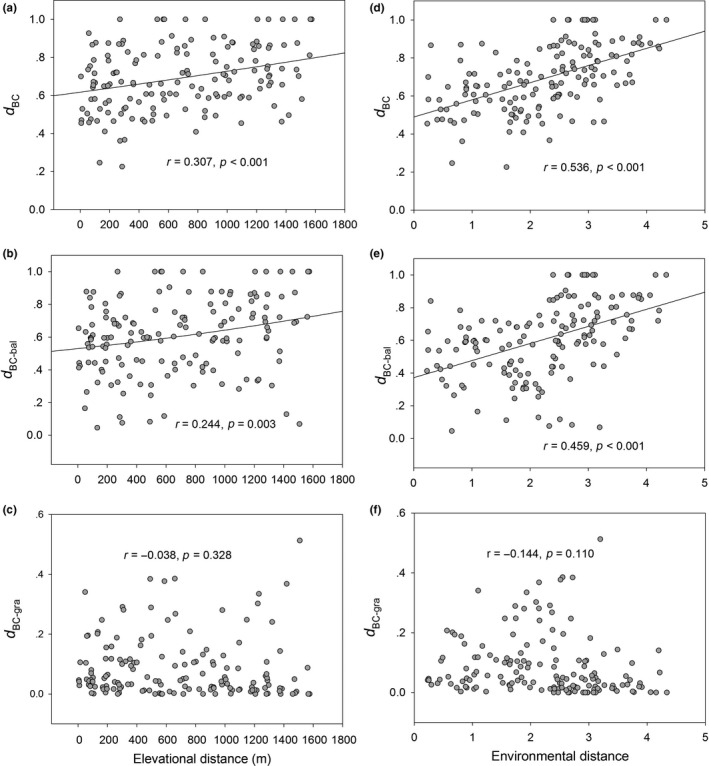
Relationship between biotic dissimilarity of soil enchytraeids and elevation change in the Changbai Mountain, as measured with the Bray–Curtis dissimilarity (*d*_BC_), its balanced‐variation (*d*_BC‐bal_), and abundance‐gradient (*d*_BC‐gra_) components. The coefficient of determination (*r*) and significance (*p*, computed using Mantel tests) of each relationship are shown

Both *d*_BC_ and *d*_BC‐bal_ significantly increased with elevational and environmental distances, with stronger correlations with environmental distance than elevational distance (Figure 1a,b,d,e, Table 2), but *d*_BC‐gra_ was not related to the two distances (Figure 1c,f). Partial Mantel tests detected significant pure effects of change in the environment (when controlling for elevational distance) on *d*_BC_ and *d*_BC‐bal_, whereas the pure effects of elevational distance were all insignificant on *d*_BC_, *d*_BC‐bal_, and *d*_BC‐gra_ (Table 2).

**Table 2:** Results of Mantel and partial Mantel tests for the correlation between biotic dissimilarity of soil enchytraeids (*d*_BC_: Bray–Curtis dissimilarity; *d*_BC‐bal_: balanced variation; *d*_BC‐gra_: abundance gradient) and elevational distance, and environmental distance (Euclidean)

	Elevation	Env	Elev–Env	Env–Elev
*d*_BC_	0.307***	0.536***	0.049	0.464***
*d*_BC‐bal_	0.244**	0.459***	0.014	0.402***
*d*_BC‐gra_	−0.038	−0.144	0.041	−0.145

Notes

Elev–Env: the effects of elevational distance on dissimilarity while controlling for environmental distance; Env–Elev: the effects of environmental distance on dissimilarity while controlling for elevational distance.

Shown are Mantel and partial Mantel correlations and their statistical significances:

** *p* < 0.01,

*** *p* < 0.001.

**Figure 2:**
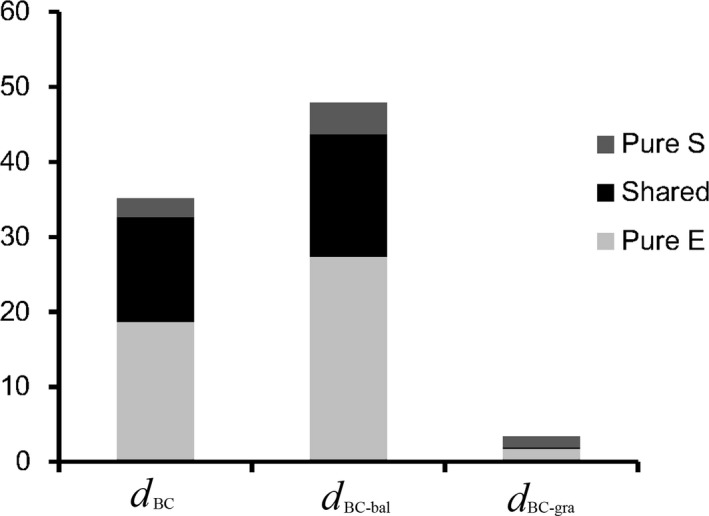
Percentage of explained variation in biotic dissimilarity of soil enchytraeids (Bray–Curtis dissimilarity, *d*_BC_; balanced variation, *d*_BC‐bal_; and abundance gradient, *d*_BC‐gra_) attributed to spatial factors (Pure S), shared fraction (Shared), and environmental factors (Pure E)

Considering the effects of spatial and environmental factors on the overall dissimilarities of enchytraeid assemblages and its two components, forward selection showed that the same spatial (PCNM1 and PCNM4) and environmental (soil temperature, litter depth, and organic nitrogen) variables were significant in controlling *d*_BC_ and *d*_BC‐bal_, but different significant predictors (PCNM3 and soil temperature) were retained for *d*_BC‐gra_ (Table 3). For *d*_BC_, *d*_BC‐bal_, and *d*_BC‐gra_, a total of 35.1%, 47.8%, and only 3.4% of the variations were explained, respectively. For *d*_BC_, variation partitioning analysis showed 18.7% explained by the pure environmental component, 14.0% by the spatially structured environmental effect (i.e., shared), and 2.4% by pure spatial filters, respectively. For *d*_BC‐bal _and *d*_BC‐gra_, these values were 27.3%, 16.3%, and 4.2%, and 1.7%, 0.3%, and 1.4%, respectively (Figure 2).

**Table 3:** Results of distance‐based redundancy analysis (dbRDA), giving the relative influence of selected environmental and spatial variables on Bray–Curtis dissimilarity (*d*_BC_), balanced‐variation (*d*_BC‐bal_), and abundance‐gradient (*d*_BC‐gra_) matrices

Variable	Adj. *R* ^2^	Pseudo‐*F*	*p*
*d*_BC_			
Environmental			
Soil temperature	0.170	4.480	<0.001
Litter depth	0.251	2.727	0.014
Organic nitrogen	0.327	2.689	0.010
Spatial			
PCNM1	0.111	3.132	0.002
PCNM4	0.165	2.020	0.051
*d*_BC‐bal_			
Environmental			
Soil temperature	0.218	5.746	<0.001
Litter depth	0.333	3.739	0.013
Organic nitrogen	0.437	3.781	0.011
Spatial			
PCNM1	0.136	3.681	0.006
PCNM4	0.205	2.385	0.062
*d*_BC‐gra_			
Environmental			
Soil temperature	0.020	1.347	0.035
Spatial			
PCNM3	0.016	1.280	0.053

## 4 DISCUSSION

Compared to most taxa of Clitellata, the taxonomy of enchytraeids is still less resolved, which hinders their extensive application in soil biodiversity assessment and environmental monitoring programs (Römbke et al., 2013; Schmelz, Niva, Römbke, & Collado, 2013). To our knowledge, our study is the first worldwide to investigate the beta diversity associated with its two components (species substitution and subset) in soil enchytraeids. We found a high level of overall abundance‐based beta diversity (ca. 70% on average) of soil enchytraeid assemblages across the Changbai Mountain, with the dominance (85%) of taxonomic substitution component (*d*_BC‐bal_) compared to *d*_BC‐gra_ (15%). This finding indicates that the enchytraeid assemblage heterogeneity was largely due to the substitution of individuals of some species by the same or similar numbers of individuals of other species.

The dominance of the turnover component over nestedness was often reported in undisturbed or lightly disturbed ecosystems (Angeler, 2013; Ding et al., 2017; Svenning, Fløjgaard, & Baselga, 2011), which highlighted that deterministic environmental filtering processes, related to species replacement between different conditions, could be attributed to historical and climatic processes or large‐scale environmental changes (Heino & Tolonen, 2017; Nunes, Braga, Figueira, Siqueira Neves, & Fernandes, 2016). Similar factors also drove the pattern of abundance‐based enchytraeid beta diversity in our study. Due to a series of environmental changes (e.g., temperature, soil moisture, litter depth) from the foot to summit of the Changbai Mountain, the community composition of Enchytraeidae was quite different among five typical forest types (Lian et al., 2011), for example, one of the dominant genera, *Bryodrilus*, mainly occurred in spruce–fir forest; *Fridericia* peaked in hardwood forest; and *Mesenchytraeus* was frequently found in high‐altitude environment with low temperature and rich organic matter. Furthermore, the pristine environment and suitable environmental conditions (e.g., abundant litter and organic matter) provide growth and reproduction advantages for these soil annelids, resulting in low differences in richness and abundance between sites at different elevations, that is, the low value of *d*_BC‐gra_.

Distance decay of enchytraeid community similarity along elevational and environmental gradients was significant for total dissimilarity and substitution, but insignificant for the subsets component. This result showed that the two antithetic components responded markedly differently to elevational and environmental distances. It is well documented that community similarity decreases as the between‐site distances increases; however, most recent studies were based on unpartitioned incidence‐ or abundance‐based dissimilarity (Teittinen et al., 2016). According to several such recent studies concerning the partitioning of overall beta diversity, inconsistent or contrasting results were obtained for the distance‐decay relationships in turnover (or substitution) and nestedness (or subsets) components (Baselga, 2013; Boieiro et al., 2013; Heino & Tolonen, 2017). In the present study, we also found Bray–Curtis dissimilarities along elevational and environmental distances were largely due to the substitution component rather than abundance‐gradient component. Therefore, the partitioning of the substitution and subsets components would be crucial for understanding the underlying diverse phenomena and their driving mechanisms behind these patterns in dissimilarity, as the substitution and loss of individuals reflect completely different ecological processes (Baselga, 2013; Baselga & Leprieur, 2015).

Based on distance‐based analysis (Mantel tests and dbRDA), the relationships between biological dissimilarity and local environmental gradient were consistently stronger compared with elevational and/or horizontal gradient, indicating the among‐site differences in community composition were more strongly controlled by effects of environmental filtering than spatial structuring. Such findings are in contrast with recent studies showing that pure elevational effects were more important than pure environmental effects on total dissimilarity of diatoms (Teittinen et al., 2016) and turnover rates of bacteria, diatoms, and macroinvertebrates (Wang et al., 2012).

In regard to the Changbai Mountain, however, some previous studies highlight the significant role of environmental factors on patterns of soil bacterial (Shen et al., 2013) and plant (Yuan et al., 2011) communities along elevational gradients. Environmental filtering is usually more important for soil communities in temperate regions than in the tropics, where spatial processes show stronger effect, as species in harsh habitat conditions (i.e., in temperate or arctic regions) are usually more common and have a higher dispersal ability than species in the tropics (Myers et al., 2013; Qian & Ricklefs, 2007).

Although information on dispersal abilities of enchytraeids is still lacking, rare species (occurring at one or two sites) of enchytraeids are really few in the studied region (Lian et al., 2011). Moreover, in well‐protected forest ecosystems, significant stochastic but weak environment filtering processes are usually observed at a fine spatial scale due to low level of habitat heterogeneity, and the effects of environmental control become stronger at a large spatial scale associated with higher differences in habitat conditions (Cao et al., 2016). On the studied mountain, there were considerable changes of environment among different forest zones from the foot to the summit of the mountain. These may be the reasons why we observed a stronger effect of environmental processes on structuring beta diversity of soil enchytraeids.

Temperature, litter depth, and organic nitrogen were determined as important predictors of beta diversity of soil enchytraeids. These variables have been identified as significant correlates to soil potworm assemblages worldwide (Briones et al., 2007; Cole et al., 2002) and in the Changbai Mountain in particular (Lian et al., 2011). Thermal regime, food resources, and habitat conditions can affect the distributions of enchytraeid species with different environmental preferences and physiological tolerances. For example, most species of *Mesenchytraeus* and *Chamaedrilus* (the original genus *Cognettia* in Lian et al., 2011) found on the Changbai Mountain typically occur in low‐temperature (high altitude or latitude region), permanently humid habitats rich in organic matter, whereas some thermophilous taxa (such as *Fridericia unisetosa*, *Enchytraeus athecatus*, *Hemifridericia parva,* and *Hemienchytraeus stephensoni*) mainly present in regions with high soil temperature, and relatively low levels of moisture and litter. The key effects of these variables on enchytraeid distributions are pertaining to the environmental filtering perspective of metacommunity theory (Leibold et al., 2004). Furthermore, it is noteworthy that the same variables were important for the overall beta diversity and substitution component of enchytraeid communities.

Contrary to overall beta diversity and its substitution component, the subsets component was poorly predicted by spatial and environmental gradients, again implying the substitution and subsets components of beta diversity derive from completely different processes. Overall, over half of the observed variation in soil Enchytraeidae assemblages in the Changbai Mountain remained unexplained, especially for the abundance‐gradient component of beta diversity. This may be attributed to methodological limitations of the dispersal‐based approaches in our study (e.g., mountain barrier and possible differences in dispersal ability of soil enchytraeids were not included) and the effects of unmeasured environmental variables (e.g., resource availability and interactions with other soil organisms).

In conclusion, our study showed that abundance‐based beta diversity of the Changbai Mountain soil enchytraeids is mainly determined by substitution of individuals among sites. Environmental filtering plays a more important role in explaining overall dissimilarity and its substitution component, indicating that niche‐based processes are determinant factors in shaping assemblage patterns of potworms on this mountain. Moreover, our findings highlight the completely different driving mechanisms of substitution and subsets components of beta diversity; thus, the two antithetic components should be jointly assessed in future beta diversity studies.

## CONFLICT OF INTEREST

None declared.

## AUTHOR CONTRIBUTIONS

X. J. and Z. X. conceived the ideas and led the writing. J. C. prepared the data. X. J. performed the analyses. Z. X. improved the ideas and analyses. All authors gave the final approval for publication.

## Data Availability

Data available from the Dryad Digital Repository: https://doi.org/10.5061/dryad.vc7cf5m.
